# The RNA cargo in small extracellular vesicles from chicken eggs is bioactive in C57BL/6 J mice and human peripheral blood mononuclear cells *ex vivo*

**DOI:** 10.3389/fnut.2023.1162679

**Published:** 2023-04-14

**Authors:** Deborah Fratantonio, Javaria Munir, Jiang Shu, Katherine Howard, Scott R. Baier, Juan Cui, Janos Zempleni

**Affiliations:** ^1^Department of Nutrition and Health Sciences, University of Nebraska, Lincoln, NE, United States; ^2^School of Computing, University of Nebraska, Lincoln, NE, United States

**Keywords:** bioavailability, chicken eggs, extracellular vesicles, gene expression, miRNA, spatial learning and memory

## Abstract

Small extracellular vesicles (sEVs) and their RNA cargo in milk are bioavailable in humans, pigs, and mice, and their dietary depletion and supplementation elicits phenotypes. Little is known about the content and biological activity of sEVs in foods of animal origin other than milk. Here we tested the hypothesis that sEVs in chicken eggs (*Gallus gallus*) facilitate the transfer of RNA cargo from an avian species to humans and mice, and their dietary depletion elicits phenotypes. sEVs were purified from raw egg yolk by ultracentrifugation and authenticated by transmission electron microscopy, nano-tracking device, and immunoblots. The miRNA profile was assessed by RNA-sequencing. Bioavailability of these miRNAs in humans was assessed by egg feeding study in adults, and by culturing human peripheral blood mononuclear cells (PBMCs) with fluorophore-labeled egg sEVs *ex vivo*. To further assess bioavailability, fluorophore-labeled miRNAs, encapsulated in egg sEVs, were administered to C57BL/6 J mice by oral gavage. Phenotypes of sEV RNA cargo depletion were assessed by feeding egg sEV and RNA-defined diets to mice and using spatial learning and memory in the Barnes and water mazes as experimental readouts. Egg yolk contained 6.30 × 10^10^ ± 6.06 × 10^9^ sEVs/mL, which harbored eighty-three distinct miRNAs. Human PBMCs internalized sEVs and their RNA cargo. Egg sEVs, loaded with fluorophore-labeled RNA and administered orally to mice, accumulated primarily in brain, intestine and lungs. Spatial learning and memory (SLM) was compromised in mice fed on egg sEV- and RNA-depleted diet compared to controls. Egg consumption elicited an increase of miRNAs in human plasma. We conclude that egg sEVs and their RNA cargo probably are bioavailable. The human study is registered as a clinical trial and accessible at https://www.isrctn.com/ISRCTN77867213.

## Introduction

Extracellular vesicles (EVs) are detectable in most, if not all, body fluids including saliva, plasma, urine, and milk (1, 2). Five classes of EVs may be distinguished based on biogenesis, size, and cargo: apoptotic bodies, microvesicles, exosomes, exomeres, and midbody remnants (3–6). Exosomes have attracted more attention than other EVs, because they facilitate cell-to-cell communication, e.g., by transferring miRNA cargo from exosome donor cells to adjacent and distant recipient cells (4). MiRNAs have been implicated in the regulation of approximately 60% of human genes and loss of miRNA maturation in Dicer knockout mice is embryonic lethal (7, 8). In this manuscript, we will refer to exosomes as small EVs (sEVs).

Food-borne sEVs and their RNA cargo have attracted considerable attention ever since it was reported that sEVs and miRNAs do not originate exclusively in endogenous synthesis but may also be absorbed from milk in humans, pigs, and mice (9–13). Milk sEVs and their miRNA cargo meet the definition of bioactive food compounds by the National Cancer Institute, which is “A type of chemical found in small amounts in plants and certain foods (such as fruits, vegetables, nuts, oil and whole grains). Bioactive compounds have actions in the body that may promote good health. They are being studied in the prevention of cancer, heart disease, and other diseases” (14). For example, human milk contains approximately 2.18 × 10^11^ sEVs/mL that harbor more than 200 distinct miRNAs (12). Milk sEVs accumulated primarily in the small intestinal mucosa, liver, spleen, heart, kidneys, and brain (11). Dietary depletion of milk sEVs and miRNAs impaired spatial learning and memory (SLM) (11), moderately decreased muscle strength (15), decreased fecundity and postnatal survival (16), and altered neurotransmitter metabolism (L-glutamate, tryptophan and purines), and bacterial communities in the cecum in humans and mice (17), whereas supplementation with milk sEVs decreased the severity of necrotizing enterocolitis in neonate mice (13) and improved bone health in mice models of osteoporosis (18).

Here, we sought to fill the knowledge gap whether milk is unique as a source of dietary sEVs and miRNA, or whether sEVs and miRNA in other animal-borne foods also have biological activity (19). We chose chicken eggs as model because, unlike milk miRNAs, chickens (*Gallus gallus*) express miRNAs with nucleotide sequences that can be distinguished from endogenous miRNAs in humans and mice, thereby serving as endogenous tracers of dietary miRNAs (20, 21). Specifically, we pursued the following aims. (1) Determine whether chicken eggs contain sEVs and assess the miRNA content of egg sEVs. (2) Determine bioavailability of chicken miRNA in humans and whether human peripheral blood mononuclear cells (PBMCs) internalize fluorophore-labeled egg sEVs and their RNA cargo *ex vivo*. (3) Assess the bioavailability and tissue distribution of fluorophore-labeled egg sEVs and miRNAs in mice. (4) Determine whether mice fed an egg sEV and miRNA sufficient (ERS) diet perform better than mice fed an egg sEV and miRNA-depleted (ERD) diet in tests of SLM.

## Materials and methods

### Small RNA sequencing analysis of eggs

Chicken eggs were purchased from a local grocery store (Hyvee^®^). Egg yolk and albumen were separated manually. Samples (50 ug) were mixed with 5 uL of proteinase K (Thermo Fisher) and incubated at 37°C with light shaking for 60 min (22). RNA was isolated from egg yolk and albumen by using Trizol. Sequencing was done on an Illumina-Hiseq 2,500 platform with a single-end 50-basepair protocol (BGI, Inc.; Hong Kong, China) using NEBNext^®^ Multiplex Small RNA Library Prep Set for illumina^®^ Set 1. The CAP-miSeq Analysis pipeline and miRDeep2 was used for analysis (23). A minimum of five sequencing reads was used as threshold for miRNAs and only miRNAs that were detected in both yolk samples are reported in this paper. The reads were mapped to chicken miRNAs using miRbase 22 (24). A threshold of minimum five sequencing reads were used to identify egg miRNAs and miRNAs detected in both yolks were reported.

### Isolation and characterization of egg sEVs

Egg yolks were mixed with ten volumes of phosphate-buffered saline (PBS). sEVs were isolated by sequential ultracentrifugation as previously described (25). Some samples were further purified by iodixanol density gradient centrifugation (26). The protein concentration in gradient fractions was measured using the BCA Protein Assay Kit (Thermo Fisher). Fraction 6 contained the highest concentration of protein and was selected for further analysis. Fraction 6 was diluted with 10 mL PBS and centrifuged at 120,000 g at 4°C for 2 h. The sEV pellet was dissolved in 1 mL PBS and characterized following EV community standards (27). In immunoblots, CD63, TSG101 and Alix were used as positive markers for extracellular vesicles, while integrin β1 was used as marker for microvesicles (28). The following antibodies and dilutions were used: mouse anti-Alix (Abcam, ab117600, Cambridge, United Kingdom, 1000-fold dilution), rabbit anti-TSG101 (Abcam, ab30871, 100-fold dilution), mouse anti-CD63 (sc-5,275 Santa Cruz, 1,000-fold dilution), and rabbit anti-integrin β1 (cat. no. 4706 Cell Signaling, Leiden, The Netherlands; 1,000-fold dilution). IRDye^®^ 680RD goat anti-rabbit IgG and IRDye^®^ 800CW goat anti-mouse IgG (LI-COR, 10,000-fold dilution) were used as secondary antibodies. Transmission electron microscopy (Hitachi H7500) and nanoparticle tracking analysis (NTA, Nanosight NS300; Malvern Instruments Ltd., United Kingdom) were conducted as previously described.

### MiRNA bioavailability in humans

Five apparently healthy men, ages 26–35 years, consumed four hard-boiled eggs within less than 10 min. Blood was collected in EDTA tubes before and at timed intervals after egg consumptions. Subjects did not consume poultry products 24 h before study began until after the last sample was collected. Plasma and Peripheral Blood Mononuclear Cells (PBMCs) were separated using density centrifugation (Histopaque-1,077^®^) as previously described (29). RNA was isolated from plasma by using Trizol and analyzed by RNA-sequencing analysis (BGI, Inc.; Cambridge, MA, United States) as described above.

RNA was extracted from PBMCs by using the RNAeasy Mini Kit (Qiagen). mRNAs were analyzed by RNA-sequencing analysis (paired-end 150-bp reads; Illumina HiSeq 2,500) in the DNA Sequencing Core at the University of Nebraska Medical Center (Omaha, NE) and selected miRNAs were analyzed by quantitative reverse transcriptase PCR (qRT-PCR) (Supplementary Table S1) (30). All human research in this paper was approved by the Institutional Review Board in the University of Nebraska-Lincoln (protocols no. 14585 and 16566).

### Internalization of sEVs and RNA cargo by human PBMCs *ex vivo*

PBMCs were cultured in RPMI-1640 media supplemented with exosome-depleted fetal bovine serum. Exo-Glow labeled sEVs (2.5 × 10^12^) were added to PBMC cultures (10^6^ cells in 3 mL media) from four subjects (ages 26–35 years) and cultured at 37°C for 9 h. Controls were cultured with unlabeled sEVs. Extracellular sEVs were removed by washing PBMCs in PBS (1,500 g, 10 min). The final PBMC pellet was resuspended in 3 mL PBS and fluorescence was measured using an EVOS FL Cell Imaging System (AMG, United States).

### RNA cargo distribution studies in mice

We used the following strategies for assessing the bioavailability distribution of egg sEV RNA in mice: In the first strategy, the entire pool of single-stranded RNA in egg sEVs was labeled with Exo-Glow following the manufacturer’s recommendations (Exo-Glow Red kit, System Biosciences, Inc., EXOR100A-1). Exo-Glow labeled sEVs were administered to C57/BL6 age 6 weeks (Jackson Laboratories, stock number 000664) by oral gavage (1 × 10^12^/g body weight). Controls received unlabeled sEVs and free Exo-Glow. Note that the vendor has replaced the Exo-Glow Red kit with a new kit that labels polyA tails and therefore cannot be used to label miRNAs. Tissues were harvested 12 and 24 h after gavage, rinsed and perfused with PBS, and fluorescence was assessed in a LI-COR Odyssey^®^ CLx. In another strategy, sEVs were loaded with two miRNAs found in egg yolk (Supplementary Table S2), i.e., synthetic IRDye-labeled miR-9 and miR-29a (IDTDNA, Inc). The miRNAs were chosen because they are implicated in SLM (31, 32). miR-9 is associated with neuronal development and miR-29 has been previously implicated in synapse formation and plasticity (33, 34). sEVs were loaded with IRDye-labeled miRNAs and extra-exosomal miRNAs were removed as previously described (10). Loaded sEVs were used within 2 h of preparation. sEVs (10^12^ sEVs /per g body weight) were administered by oral gavage to C57/BL6 mice age 6 weeks. Controls received unloaded sEVs. Tissues were harvested 12 h after gavage, rinsed and perfused with PBS, and fluorescence was assessed in a LI-COR Odyssey^®^ CLx. All animal experiments were approved by the Institutional Animal Care Program at the University of Nebraska-Lincoln (protocol 1229).

### Gene expression in the murine hippocampus

We conducted an egg sEV and miRNA feeding study to assess whether dietary depletion elicits gene expression changes in the hippocampus, i.e., a region in the brain that is crucial for SLM (35, 36). The pelleted rodent diets represent a variation of our milk sEV and RNA-defined diets and are based on the AIN-93G formulation (37, 38). Briefly, in the egg sEV and RNA-sufficient (ERS) diet, the equivalent of four egg yolks consumed by an adult per day is substituted for 80 g lyophilized yolk (Supplementary Table S3). In the egg sEV and RNA-depleted (ERD) diet, the egg yolks were ultrasonicated for 90 min (VWR Aquasonic 250 T) and incubated at 37°C for 60 min prior to pelleting. Ultrasonication led to a decrease of 62.1 ± 3.0, 81.3% ± 1.7, 82.4% ± 1% and 92.1 ± 1.5% of miR-30a-5p, miR-92-3p, miR-181-5p and miR-221-3p, respectively, compared to non-ultrasonicated yolks. Previous studies showed that ultrasonication leads to an irregular sEV surface morphology and a decrease in sEV count (37). All other ingredients are identical in ERS and ERD diets. Male and female mice were fed experimental diets from age 3 to 9 weeks with free access to food and water (12-h light–dark cycle; 22°) when ERS/ERS and ERD/ERD pairs were mated. Offspring continued on parental diets until age 22 weeks when hippocampi were harvested.

RNA was purified using the miRNAeasy Mini Kit (Qiagen). RNA integrity was confirmed in the DNA Sequencing Core at the University of Nebraska Medical Center by using a Fragment Analyzer Automated CE System (Advanced Analytical Technologies, Inc.). Libraries were prepared by using the TrueSeq RNA Library Prep Kit v2 (Illumina, Inc.) and sequenced using an Illumina HiSeq 2,500 platform and a 150 base-pair paired-end protocol (RNA-seq). The sequencing reads were annotated to the mouse reference genome [GRCm38, mm10] using RSEM (39).

### Spatial learning and memory

Barnes and Morris water mazes were used to assess SLM. Tests were performed in C57BL/6 breeders fed ERS or ERD diets (ERS x ERS and ERD x ERD), and in F1 pups born to ERS and ERD parents and fed parental diets (*n* = 8). In the Barnes maze, breeders were studied at age 12 weeks and F1 offspring was studied at age 16 weeks as previously described (40). Mice were trained three times per day for six consecutive days, followed by a performance test on day 7. The ANY-maze video tracking system (Stoelting Co., Wood Dale, IL, United States) was used to record the time until mice entered the escape hole. The Morris water maze tests were conducted as previously described at a water temperature of 26°C (41).

### Statistical analysis

Data variances were homogenous as per Bartlett’s test. Effect of eggs consumption on mRNA abundance in PBMCs was analyzed by the paired, two-sided t-test. miRNA concentrations in plasma were analyzed by the Wilcoxon signed-rank test. GraphPad Prism 6 (GraphPad Software Inc.) was used for statistical analyses. Differentially expressed transcripts were identified by DESeq2 (42) using the paired-sample design and multiple test correction (adjusted value of *p* < 0.05 for multiple comparisons using Benjamini’s method). Differences were considered statistically significant at *value of p* < 0.05.

## Results

### sEV in eggs

**Figure 1:**
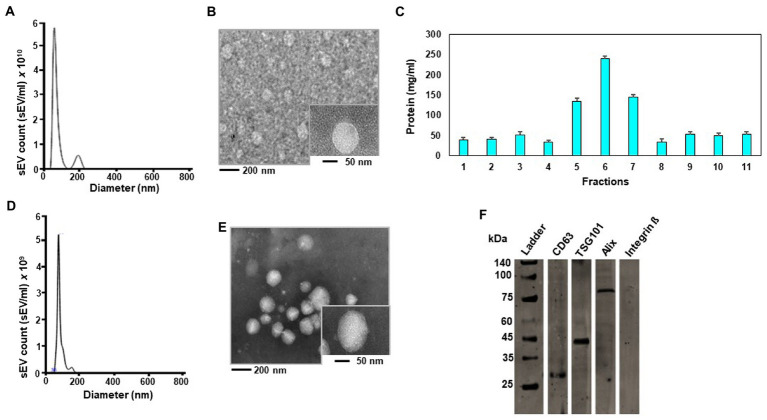
Authentication of small extracellular vesicles (sEVs) from chicken egg yolk. Size distribution of sEVs isolated by ultracentrifugation without subsequent iodixanol gradient centrifugation analyzed in a Nanosight NS300 instrument **(A)**. Transmission electron microscope (TEM) image of sEV suspension isolated by ultracentrifugation without subsequent iodixanol gradient centrifugation. The large-field image was obtained at 20,000-fold magnification and the insert depicts an sEV at 50,000-fold magnification **(B)**. Protein concentration in iodixanol gradient fractions **(C)**. Size distribution of sEVs in iodixanol gradient fraction 6 **(D)**. TEM image of sEVs in iodixanol gradient fraction 6. The large-field image was obtained at 20,000-fold magnification and the insert depicts an sEV at 50,000-fold magnification **(E)**. Immunoblot of chicken egg sEVs in iodixanol gradient fraction 6. Samples were run on the same gel. Lanes were cut for probing with antibodies and electronically re-assembled. Proteins were detected as follows: CD63 at 26 kDa, TSG101 at 44 kDa, Alix at 96 kDa and no Integrin ᵝ at 130 kDa **(F)**.

When sEVs were isolated by sequential centrifugation, the concentration was 6.30 × 10^10^ ± 6.06 × 10^9^ sEVs per mL yolk with a diameter of 110.0 ± 20.5 nm (mean ± SD) (Figure 1A). Consistent with the previous studies of sEVs (11), egg sEVs showed a spherical shape (Figure 1B). Iodixanol density centrifugation was used for further purification of egg sEVs. Column fraction 6 contained 7.90 × 10^9^ ± 0.19 × 10^3^ sEVs (Figure 1C). The diameter measured 99.5 ± 30.1 nm (mean ± SD) for sEVs purified by a combination of ultracentrifugation and iodixanol gradient (Figure 1D), which is the size expected for these vesicles (11). TEM analysis revealed a spherical shape (Figure 1E). Immunoblots detected exosome markers (CD63, TSG101 and Alix) in egg sEVs, whereas a microvesicle marker (integrin β1) was not detectable (Figure 1F).

### miRNAs in egg sEVs

Two eggs yielded 0.141 and 0.319 ng RNA per μL yolk. Eighty-three miRNAs were identified in both yolks by RNA-sequencing analysis. Out of these eighty-three miRNAs, fifty-six were common in both yolk samples (Supplementary Table S2). Two eggs yielded only 0.012 and 0.017 ng RNA per μL albumen, which was insufficient for RNA-sequencing analysis.

### Bioavailability of egg miRNAs in humans

**Figure 2:**
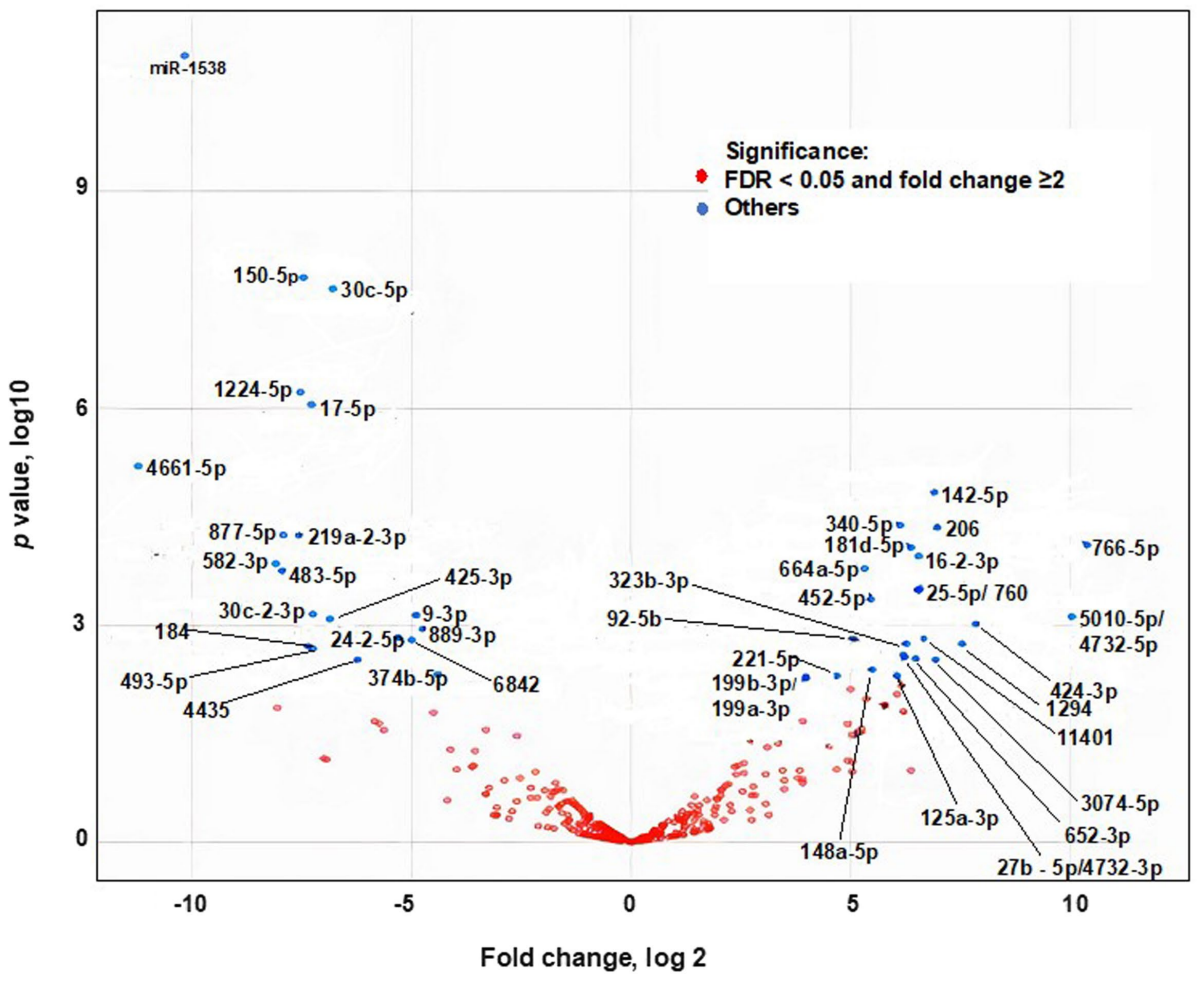
Effect of egg consumption of miRNAs expression in human plasma. Blue dots denote miRNAs for which the expression differs at least 2-fold before compared to 9 h after egg consumption (False Discovery Rate adjusted Value of *p* < 0.05).

Egg consumption elicited postprandial changes in plasma miRNA levels in dose–response studies in adults. Plasma miRNAs were analyzed before and 9 hours after consuming two to four eggs (*n* = 5). One hundred-seventeen miRNAs were identified by RNA-sequencing analysis. Twenty-six miRNAs were upregulated after egg consumption; the others were downregulated or remained unchanged (Figure 2, Supplementary Table S4). The nucleotide sequences of 75 miRNAs detected in plasma 9 h after egg consumption are identical in humans and chickens (43). Thirty-one miRNAs have near-identical sequences in chickens and humans with short extensions (1–4 base pairs) at the 5′-end or the 3′-end. Eleven miRNAs have nucleotide sequences that differed by 1 bp in chickens and humans.

These eleven chicken specific miRNAs were below detection limit in human plasma after egg meals (low RPKM). Their low abundance in plasma was attributed to their low abundance in egg yolk, e.g., miR-146c-5p (Supplementary Table S2).

### miRNA levels and mRNA expression in human peripheral blood mononuclear cells

**Figure 3:**
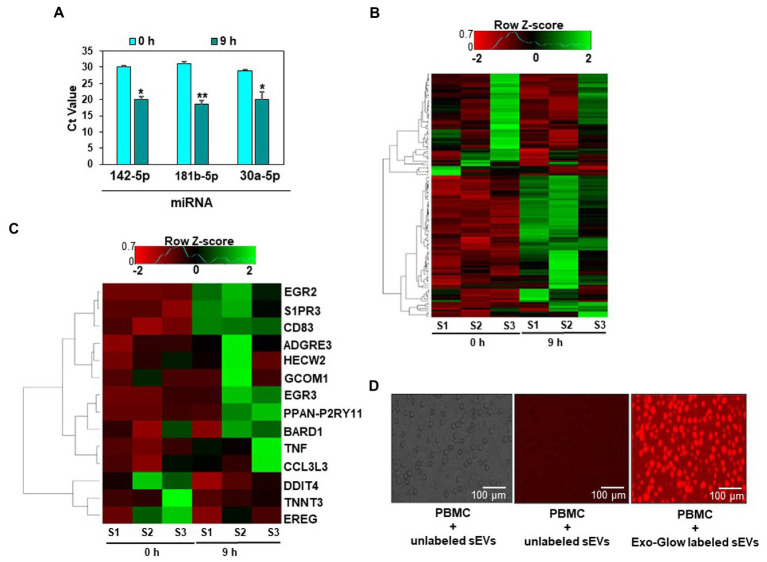
Egg small extracellular vesicles (sEVs) deliver miRNAs to human peripheral blood mononuclear cells (PBMCs). Expression of miRNAs detected in PBMCs 9 h after egg consumption. Data are presented as means ± standard deviations. ^*^Value of *p* <0.05 (*n* = 5) for Student’s paired *t*-test **(A)**. Heat map of the expression data of all genes in PBMCs **(B)**. Heat map of fourteen genes for which the expression was at least 2-fold or more different after the egg meal. For each time point, samples (*n* = 3) were collected from the same subjects before and after the egg meal **(C)**. Uptake of Exo-Glow labeled RNAs, encapsulated in egg sEVs by human PBMCs **(D)**.

Egg consumption caused an increase in miRNA levels in PBMCs, and the increase was associated with changes in gene expression *in vivo*. We isolated PBMCs from human subjects before and after consumption of four eggs and analyzed the levels of three miRNAs by qRT-PCR. The levels of miR-142-5p, miR-181b-5p, and miR-30a-5p were more than 2-fold higher 9 hours after egg consumption compared to before consumption (Figure 3A).

Eighty-eight genes were differentially expressed in PBMCs before and after egg consumption, as per RNA-sequencing analysis. For 14 mRNAs the difference was 2-fold or greater (Figures 3B,C, Supplementary Table S5). Using all differentially expressed mRNAs as input, twenty Kyoto Encyclopedia of Genes and Genomes (KEGG) pathways and two hundred and forty-one Gene Ontology (GO) terms were identified (Supplementary Table S6). mRNA-sequencing data are available in BioProject (PRJNA422156). The sEV-dependent changes in miRNA and mRNA expression *in vivo* are consistent with our observation that human PBMCs internalized Exo-Glow Red labeled egg sEVs *ex vivo* (Figure 3D).

### Egg RNA distribution in mice

**Figure 4:**
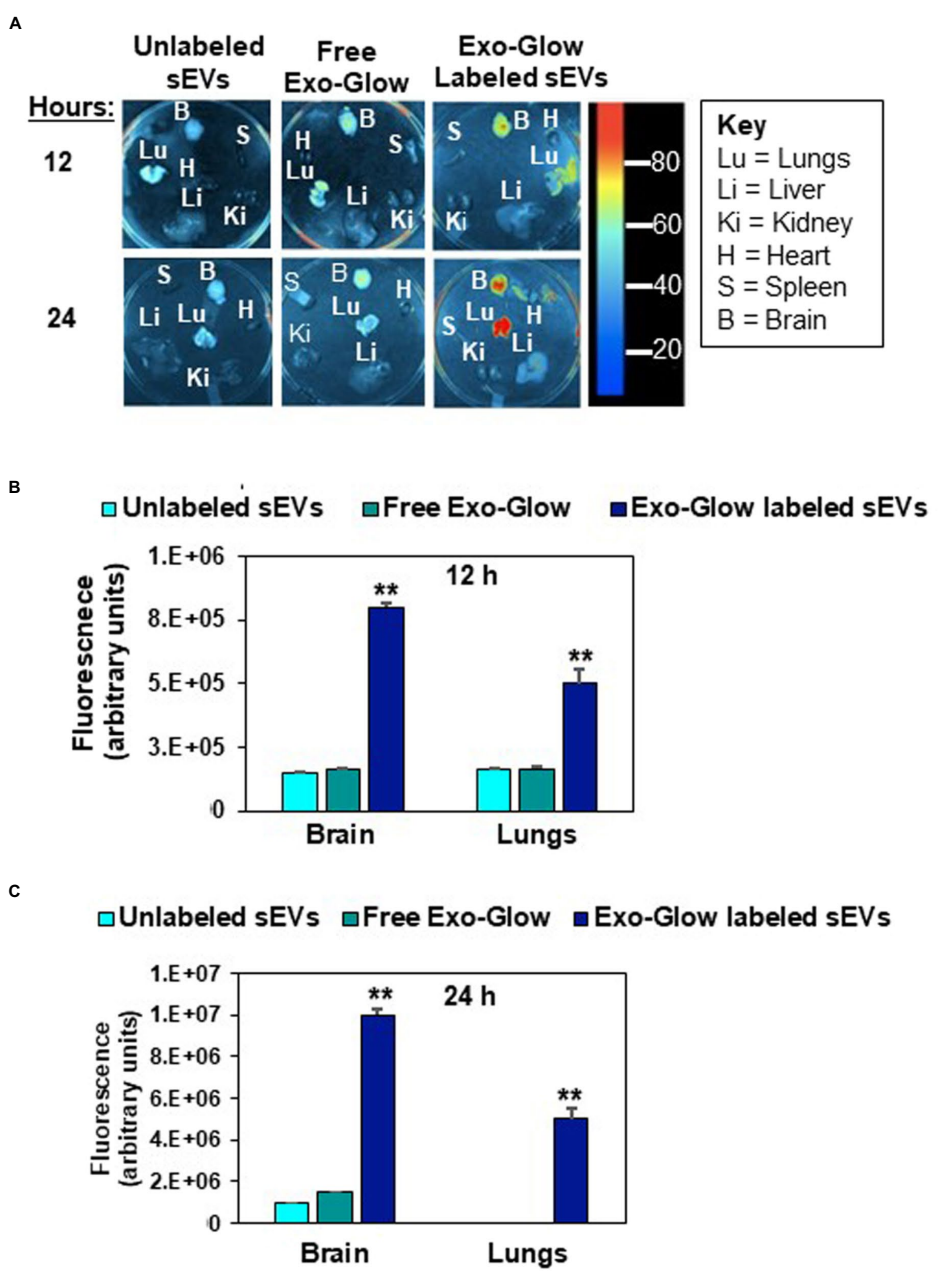
Distribution of Exo-Glow labeled small extracellular vesicles (sEVs) in C57BL/6 J mice. Free Exo-Glow, unlabeled egg sEVs and Exo-Glow labeled sEVs were administered by oral gavage. Mice were euthanized 12 h and 24 h after oral administration of sEVs **(A)**. Densitometry analysis of background-normalized fluorescence in brain and lungs 12 h **(B)** and 24 h **(C)** after oral gavage. Data were normalized by using the fluorescence from tissue-free sections in the dish. Values are mean ± SEM (*n* = 3). Data were analyzed by Mann–Whitney *U* test (^**^Value of *p* < 0.01).

**Figure 5:**
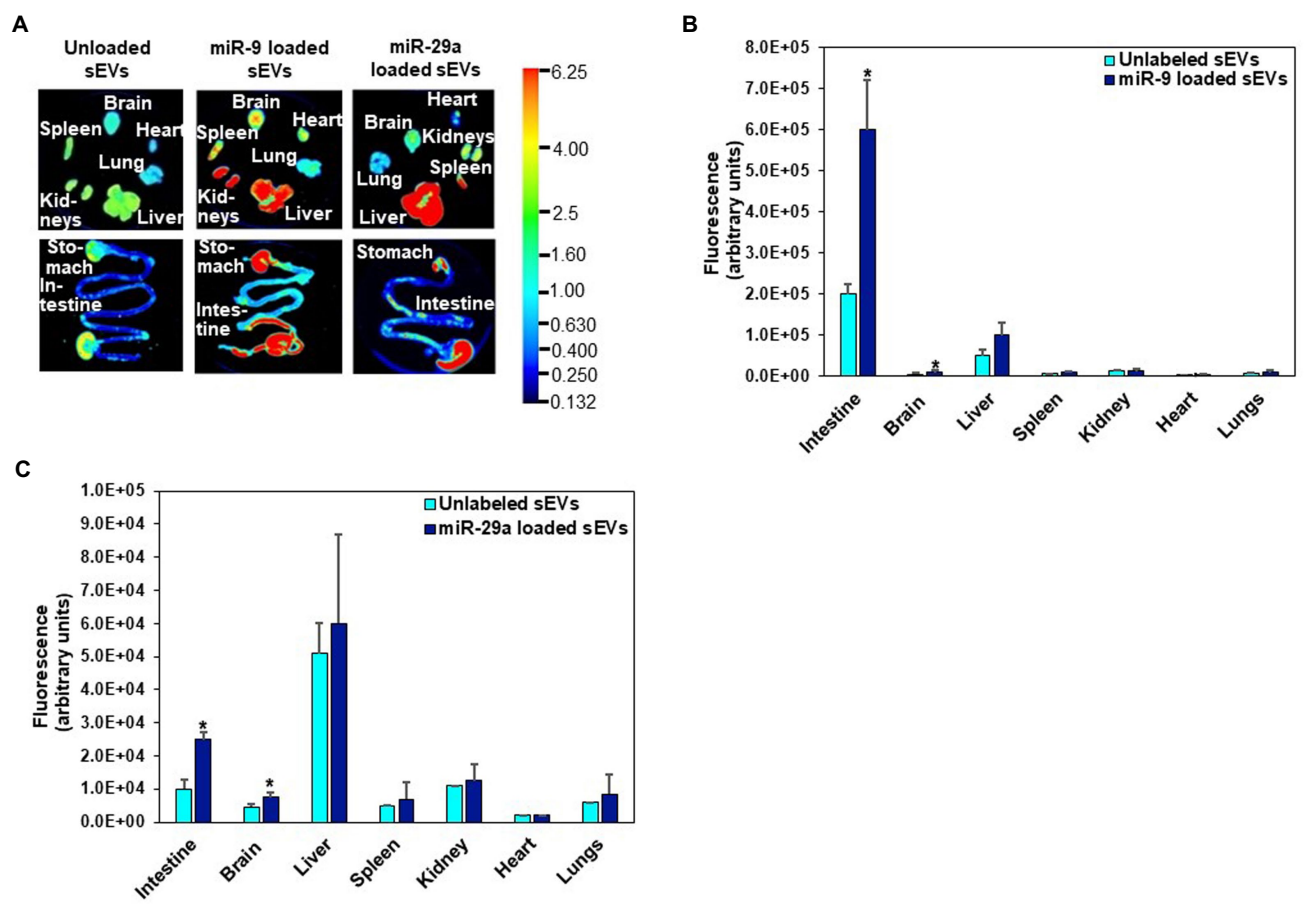
Distribution of egg sEVs loaded with IRDye-labeled miR-9 or miR-29a and delivered by oral gavage in C57BL/6 J mice **(A)**. Densitometric analysis of background-normalized fluorescence in intestine, brain, liver, spleen, kidney, heart, and lungs showing accumulation of miR-9 **(B)** and miR-29a **(C)**. Values are mean ± SEM (*n* = 3). Data were analyzed by Mann–Whitney *U* test (^*^Value of *p* < 0.05). Fluorescence is reported as arbitrary units (au) **(B, C)**. Data were normalized by using the fluorescence from tissue-free sections in the dish.

Fluorophore-labeled RNA, encapsulated in egg sEVs, was detected in the intestinal mucosa and peripheral tissues. Two RNA labeling strategies were used to assess sEV cargo distribution. In one of the strategies, the entire pool of single-stranded RNA in sEVs was labeled with Exo-Glow. Exo-Glow labeled RNA accumulated in brain and lungs when administered by oral gavage (Figures 4A–C). Using an alternative strategy, egg sEVs were loaded with IRDye-labeled synthetic miRNAs. Oral administration of the miRNA-loaded egg sEVs led to the accumulation of miRNAs (miR-9 and miR-29a) in the intestinal mucosa and brain (Figures 5A–C).

### Egg sEV-dependent gene expression changes in the brain and associated SLM phenotypes

**Figure 6:**
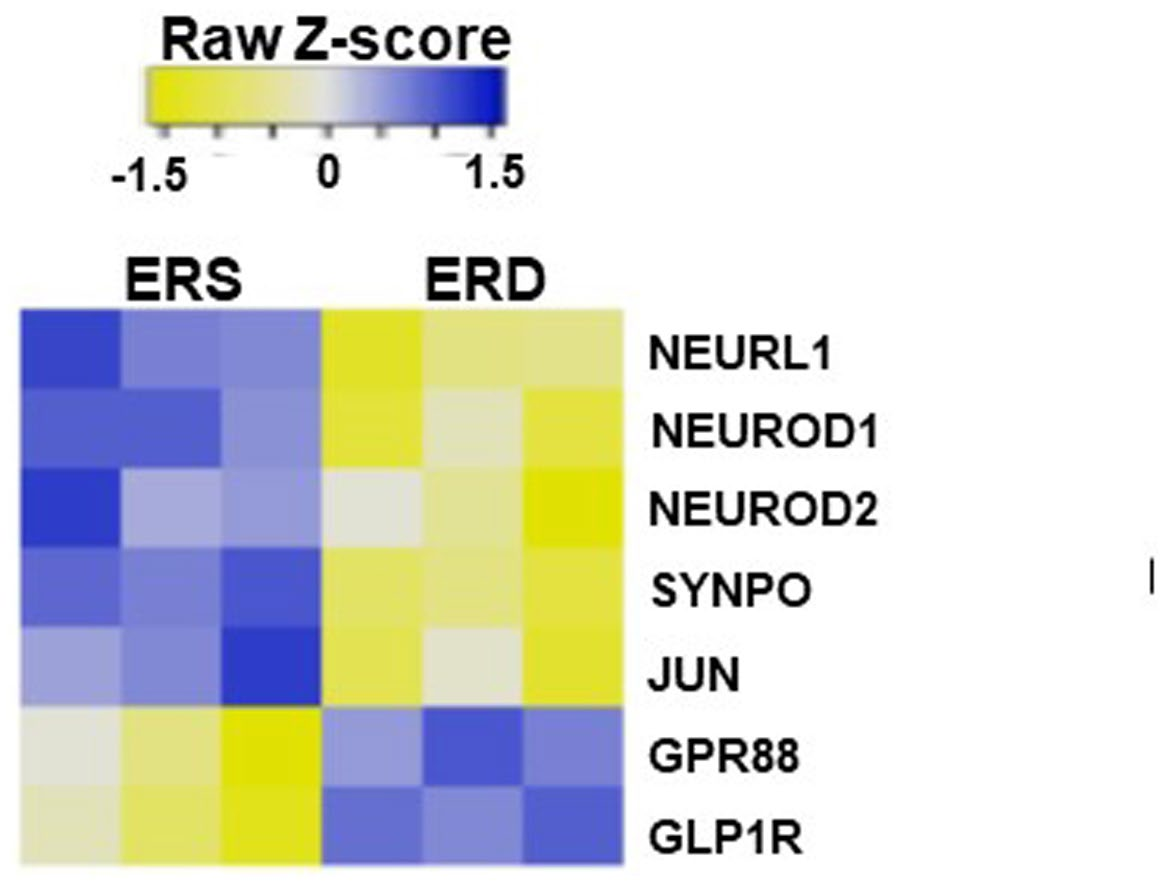
Heat map of differential expression in genes associated with spatial learning and memory in mice brain fed by sEV rich and sEV deficient diet.

The accumulation of RNA in egg sEVs in the brain was associated with changes in gene expression. When mice were fed egg sEV and RNA-deficient or egg sEV and RNA-sufficient diets, seven genes implicated in SLM were differentially expressed in the hippocampus (Figure 6), i.e., the region of the brain that regulates SLM (35, 44).

**Figure 7:**
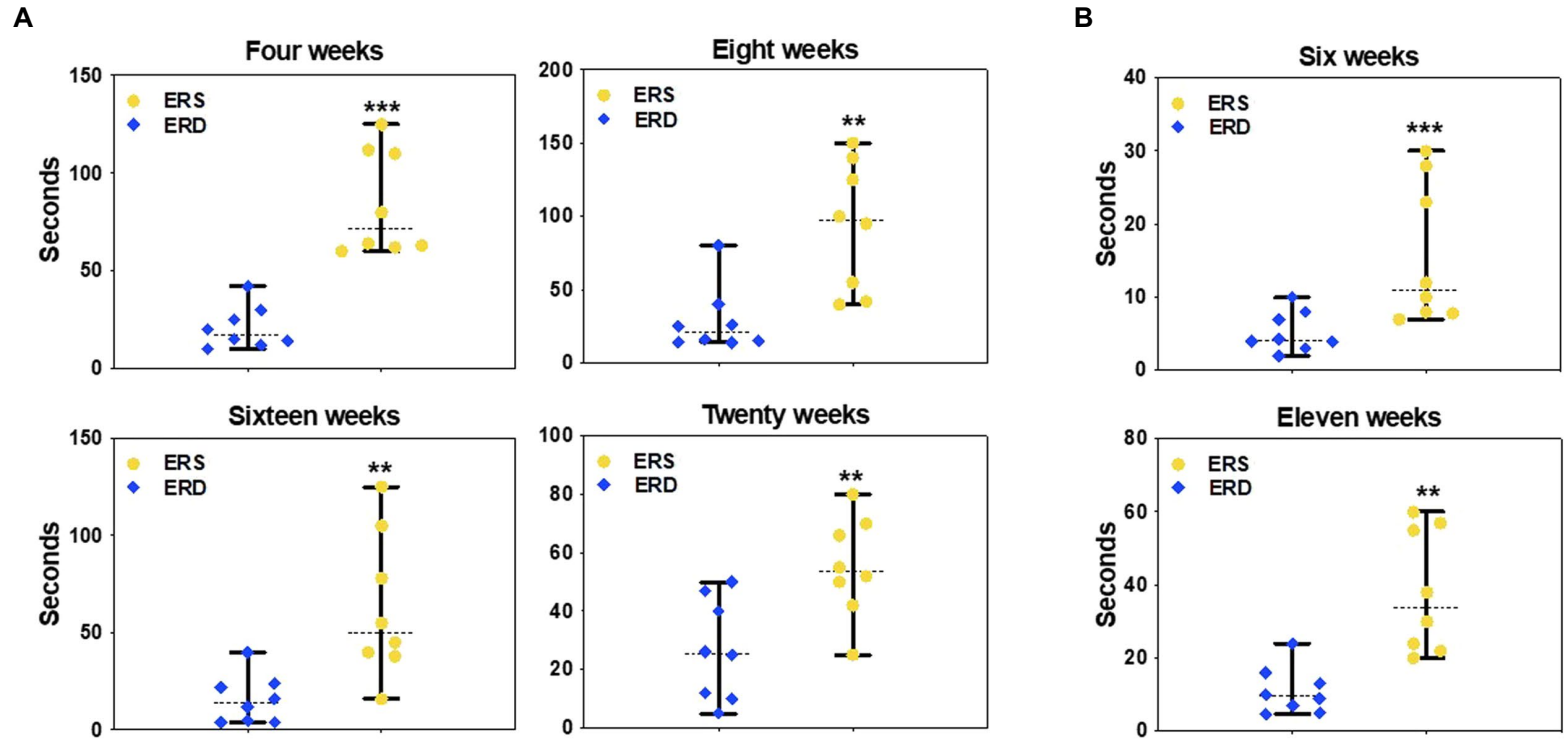
Effect of egg small EVs and RNA-sufficient (ERS) diet on spatial learning and memory in C57/BL6J mice. Latency to locate the escape platform in the Barnes maze after consuming sEV RNA rich and sEV RNA deficient diets for 4, 8, 16, and 20 weeks in C57BL/6 mice **(A)**. Latency to locate the escape platform in the Morris Water maze after consuming ERS or ERD diets for 6 and 11 weeks in C57BL/6 mice **(B)**. Data are presented as median with range (*n* = 8). The middle line denotes the median. Data were analyzed by Mann–Whitney test. Bars indicate the minimum and maximum values. ^*^Value of *p* <0.05; ^**^Value of *p* <0.01.

Dietary depletion of egg sEVs and RNA impaired SLM, assessed by Barnes maze and Morris water maze in mice. Mice fed on ERD diet took more than ten times longer to locate and enter the escape hole in a Barnes maze compared to ERS-fed mice at 4, 8, 16, and 20 weeks of feeding (Figure 7A). Morris water maze test results showed that the mice fed on ERD diet took longer to locate the hidden platform, compared to ERS-fed mice at 6 and 11 weeks (Figure 7B).

## Discussion

The presence of extracellular vesicles (EVs) in diet such as edible plant juices and milk has been the center of attention for the past few years. Studies suggest that these vesicles and their cargo (including miRNA) can be absorbed through intestine and travel *via* blood stream affecting the health of various organs through gene-regulation and ultimately influencing consumer health. Here, in this study, we explored and validated the presence of sEVs and its miRNA cargo in chicken eggs particularly in yolk. Consumption of chicken eggs led to the presence of chicken miRNA in blood sera of consumers, which potentially were absorbed through intestine. Similarity of chicken and human miRNAs indicate their potential role in regulating immune response, metabolic and vital cellular processes in human consumers.

This study provides compelling evidence that chicken egg yolk, but not albumen, contains detectable amounts of sEVs with features expected of exosomes, including morphology, size, marker proteins, and miRNA cargo. Yolk content is the sole source of nutrition for the avian embryo (45, 46) and it is reasonable to propose that yolk sEVs and their RNA cargo are crucial for normal embryonic development similar to what has been reported for milk sEVs and postnatal development in mammals (16).

The intent of this study was to assess the biological activity of egg sEVs and miRNA cargo in non-avian species. After the initial demonstration that egg yolk contains sEVs and documenting the miRNA content of the sEVs, we focused on two aspects that are crucial when assessing the relevance in nutrition, namely bioavailability and phenotypes of depletion. Evidence is strong that human PBMCs internalize egg sEVs and their RNA cargo *ex vivo*, and egg miRNAs accumulate in murine tissues, predominantly in the gastrointestinal mucosa, brain, and lungs. In contrast, we cannot state with a high degree of confidence that avian miRNAs entered human circulation. While postprandial changes in plasma miRNA levels were readily apparent in humans after egg consumption, the changes were limited to miRNAs for which the nucleotide sequence is conserved in humans and chickens. We attributed the absence of a postprandial increase in chicken-specific miRNAs in human plasma to their low content in yolk. For example, *gga*-miR-146c-5p was one of the least abundant miRNAs in yolk and not detected in human plasma. We acknowledge the limitation that we did not collect a 9-h sample from subjects who did not consume eggs, but used the baseline sample as control. Approximately a third of plasma miRNAs exhibit a diurnal variation (47).

We identified two explanations for postprandial changes in miRNA levels in plasma and PBMCs. In one scenario, egg consumption increases or decreases the endogenous synthesis of miRNAs, i.e., the miRNAs do not originate in eggs. In the other scenario, egg miRNAs are bioavailable. An alternative, not mutually exclusive, explanation is that humans absorbed miRNAs from egg sEVs, which led to changes in endogenous miRNA levels. Unfortunately, chicken-specific miRNAs are rare in egg sEVs. A limitation of the bioavailability arm of this study is that one cannot determine the effects of boiling on the content of sEVs and miRNAs in eggs because it proved impossible to purify sEVs and RNA from hard-boiled eggs (data not shown).

As for phenotypes of sEV and miRNA depletion, we focused on SLM because loss of SLM was among the strongest phenotypes of (milk) sEV and RNA depletion observed in previous studies in mice (11). In the previous studies of milk sEVs, the latency for entering the escape hole in the Barnes maze was 9-fold greater in adolescent mice fed a milk sEV-depleted diet compared to controls fed a milk sEV-sufficient diet. Diet effects were similar in this study of egg sEVs and SLM. The number of hippocampal genes that were differentially expressed in sEV and miRNA-defined diets was greater for bovine milk (219 genes) than eggs (86 genes). We speculate that the differential effects of milk and egg sEVs in the hippocampus might be due to a higher degree of evolutionary conservation in cow and mice compared to chicken and mice.

We conclude that studies of milk sEVs are a more fruitful line of investigation than studies of chicken sEVs, primarily because of the unique role of milk in infant nutrition and the use of milk sEVs in the delivery of therapeutics (48, 49). Because of the low content of chicken-specific miRNAs in eggs, we could not detect such transcripts in human plasma with satisfactory confidence. We shall point out a unique opportunity afforded by egg sEVs. Hens deposit in eggs what is crucial for early embryonic development, and it would be of interest to study the time courses of miRNA biogenesis in developing avian embryos. Such studies could lead to the identification of miRNAs that are essential during the early stages in embryonic development. Secondly, the study opens a new avenue of research in egg nutrition and its short- and long-term effects on human health.

## Data availability statement

The datasets presented in this study can be found in online repositories. The names of the repository/repositories and accession number(s) can be found at: https://www.ncbi.nlm.nih.gov/, PRJNA422156.

## Ethics statement

The studies involving human participants were reviewed and approved by Institutional Review Board in the University of Nebraska-Lincoln (protocols no. 14585 and 16566). The patients/participants provided their written informed consent to participate in this study. The animal study was reviewed and approved by Institutional Animal Care Program at the University of Nebraska-Lincoln (protocol 1229).

## Author contributions

JZ conceived the project. DF, KH, and SB collected data. JM, DF, JS, KH, and JC analyzed data. JM and DF wrote the draft manuscript. JZ wrote the final manuscript. All authors contributed to the article and approved the submitted version.

## Funding

This study was supported by the National Institutes of Health (NIH 1P20GM104320), the National Institute of Food and Agriculture (NIFA 2016–67001-25301), the Egg Nutrition Center, United States Department of Agriculture (Hatch NEB-36-087 and W4002).

## Conflict of interest

The authors declare that the research was conducted in the absence of any commercial or financial relationships that could be construed as a potential conflict of interest.

## Publisher’s note

All claims expressed in this article are solely those of the authors and do not necessarily represent those of their affiliated organizations, or those of the publisher, the editors and the reviewers. Any product that may be evaluated in this article, or claim that may be made by its manufacturer, is not guaranteed or endorsed by the publisher.
