# Supplementary material for: The RNA cargo in small extracellular vesicles from chicken eggs is bioactive in C57BL/6 J mice and human peripheral blood mononuclear cells ex vivo
Source: Front Nutr. 2023 Apr 14;10:1162679. doi: 10.3389/fnut.2023.1162679 (PMC10249500; doi:10.3389/fnut.2023.1162679)
Supplement: Supplementary file 3 [file Data_Sheet_1.PDF]

Supplemental Table 1. RT-PCR primers for miRNAs.

Amplicon	Forward Primer	Reverse Primer
**miSPIKE[1]**	5’CTCAGGATGGCGGAGCGGTCT-3’	N.A
**gga/hsa-miR-142-5p**	5’-CATAAAGTAGAAAGCACUACT-3’	N.A
**gga/hsa-miR-181b-5p**	5’-AACATTCATTGCTGTCGTGGGT-3’	N.A
**gga/hsa-miR-30a-5p**	5’-TGTAAACATCCTCGACTGGAAG -3’	N.A

[1]miRNA; N.A, not available (proprietary information, not disclosed by vendor)

Supplemental Table 2. Identified known miRNAs in chicken egg yolk through a small RNA sequencing analysis. Normalized counts represent the ratio of corresponding supporting reads and total mapped reads.

microRNAs	Normalized Counts		microRNAs	Normalized Counts		microRNAs	Normalized Counts	
	Yolk-1	Yolk-2		Yolk-1	Yolk-2		Yolk-1	Yolk-2
**gga-miR-142-3p**	154516.43	163251.09	**gga-miR-126-5p**	2890.35	2159.02	**gga-miR-132a-3p**	2267.58	
**gga-miR-375**	157814.74	152407.25	**gga-miR-92-3p**	2498.9	2250.18	**gga-miR-128-3p**	1006.37	
**gga-miR-10b-5p**	149060.29	151411.61	**gga-miR-21-5p**	3288.07	1052.4	**gga-miR-1388a-5p**	937.07	
**gga-miR-181a-5p**	36860.61	33132.13	**gga-miR-103-3p**	2824.91	1340.39	**gga-let-7d**	927.88	
**gga-let-7f-5p**	30807.1	32086.33	**gga-miR-30e-5p**	1669.28	2343.77	**gga-miR-30c-2-3p**	590.68	
**gga-miR-10a-5p**	20005.51	35323.27	**gga-let-7b**	2228.39	1063.06	**gga-miR-29a-3p**	556.6	
**gga-miR-148a-3p**	24574.65	25404.72	**gga-let-7g-5p**	2484.47	537.88	**gga-miR-29c-3p**	556.6	
**gga-let-7i**	21814.95	26656.86	**gga-miR-129-1-3p**	528.38	2400.02	**gga-miR-145-3p**	452.07	
**gga-miR-182-5p**	20704.37	23724.03	**gga-miR-142-5p**	1761.16	982.81	**gga-miR-153-3p**	213.55	
**gga-miR-27b-3p**	15833.88	24702.15	**gga-miR-16-5p**	479.98	2249.55	**gga-miR-133b**	1.25	
**gga-miR-26a-2-5p**	18360.27	21264.84	**gga-miR-338-3p**	1047.14	1462.92	**gga-miR-129-5p**		1621.77
**gga-miR-26a-5p**	18360.27	21264.84	**gga-miR-199-3p**	1345.56	740.28	**gga-miR-23b-3p**		1616.06
**gga-let-7a-5p**	20047.56	17218.46	**gga-miR-140-3p**	1562.97	451.54	**gga-miR-221-3p**		1483.49
**gga-let-7j-5p**	19914.26	17216.89	**gga-miR-200b-3p**	879.38	922.88	**gga-miR-30e-3p**		1256.07
**gga-miR-22-3p**	10322.24	14123.95	**gga-miR-107-3p**	566.53	1085.41	**gga-miR-15a**		1073.48
**gga-miR-100-5p**	11557.63	9873.72	**gga-miR-133a-3p**	1151.87	444.55	**gga-miR-31-5p**		830.31
**gga-miR-30a-5p**	8567.98	10824.41	**gga-miR-205a**	408.49	1004.53	**gga-miR-130b-3p**		698.51
**gga-miR-191-5p**	10391.64	6788.78	**gga-miR-9-5p**	832.65	574.58	**gga-miR-30a-3p**		644.04
**gga-miR-101-3p**	11000.4	5172.98	**gga-miR-183**	353.4	890.12	**gga-miR-193b-3p**		396.17
**gga-miR-125b-5p**	9381.56	3989.66	**gga-miR-133c-3p**	1150.41	1.27	**gga-miR-148b-3p**		383.73
**gga-miR-451**	2784.25	7946.32	**gga-let-7l-5p**	534.65	604.29	**gga-miR-145-5p**		352.62
**gga-miR-200a-3p**	5573	4178.61	**gga-miR-146a-5p**	0.63	1018.88	**gga-miR-212-5p**		295.1
**gga-miR-204**	5500.57	2603.06	**gga-miR-222a**	520.43	407.86	**gga-miR-130c-3p**		245.45
**gga-miR-211**	5500.57	2603.06	**gga-let-7k-5p**	571.13	30.35	**gga-miR-181a-3p**		95.74
**gga-miR-99a-5p**	3867.98	3810.37	**gga-miR-10c-5p**	511.55	18.67	**gga-miR-16c-5p**		1.65
**gga-let-7c-5p**	2763.35	4827.47	**gga-miR-215-5p**	3.66	9.14	**gga-miR-146b-5p**		1.02
**gga-miR-30d**	3320.37	2290.95	**gga-miR-126-3p**	0.63	0.76	**gga-miR-146c-5p**		1.02
**gga-miR-181b-5p**	3310.75	2197.74	**gga-miR-125b-3p**	2664.99				

Supplemental Table 3. Composition of experimental diets[1]

Ingredient	ERD[2]	ERS
		g
Cornstarch	375	375
Soy protein	163	163
Dextrinized cornstarch	147	147
Sucrose	100	100
Soybean oil (no additives)	35	35
Fiber	50	50
Mineral mix (AIN-93G-MX)	35	35
Vitamin mix (AIN-93-VX)	10	10
L-Cystine	3	3
Choline bitartrate (41.1% choline)	2.5	2.5
Tert-butylhydroquinone	0.014	0.014
Lyophilied egg yolk[3]	80	80

[1]Energy content 3.9 kcal/g

[2]ERD, Egg sEV- and RNA-depleted diet; ERS, Egg sEV- and sufficient diet.

[3]ERD diet contains egg yolk that was ultrasonicated for 90 min an incubated at 37°C. ERS diet contains egg yolk that was not ultrasonicated.

Supplementary Table 4. Plasma levels (sequencing reads) of miRNAs significantly changed by consumption of four hard boiled eggs in adults.

miRNA	Sequencing reads[1]	
	Before egg consumption	After egg consumption
hsa-miR-11401	4.7 ± 3.8	1038 ± 1551
hsa-miR-125a-3p	1.3 ± 0.6	831 ± 1386
hsa-miR-1294	168 ± 289	1280 ± 2190
hsa/gga-miR-142-5p[2,*]	182 ± 302	4245 ± 4780
hsa-miR-148a-5p	42 ± 70	1604± 1542
hsa-miR-16-2-3p[*]	843 ± 1445	3213 ± 3587
hsa-miR-181d-5p/gga-miR-181b-5p[2,*]	39 ± 43	7297 ± 10753
hsa-miR-199a-3p	1203 ± 1557	8849± 7530
hsa-miR-199b-3p	1203 ± 1557	8846 ± 7536
hsa/gga-miR-206[2,*]	5.7 ± 7.2	3069 ± 4948
hsa/gga-miR-221-5p[2]	164 ± 270	2581 ± 2305
hsa-miR-25-5p[*]	1804 ± 3112	3164 ± 3440
hsa/gga-miR-27b-5p[2]	1.3 ± 0.6	1053 ± 1766
hsa-miR-3074-5p	1.0 ± 1.7	915± 1565
hsa-miR-323b-3p	78 ± 132	1097 ± 1549
hsa-miR-340-5p[*]	445 ± 751	5006 ± 3590
hsa-miR-424-3p	62 ± 108	874 ± 1488
hsa-miR-452-5p[*]	79 ± 123	5178 ± 6103
hsa-miR-4732-3p	3.0 ± 1.0	956 ± 1477
hsa-miR-4732-5p[*]	472 ± 806	2893 ± 2367
hsa-miR-5010-5p	102 ± 174	1193 ± 2031
hsa-miR-652-3p	332 ± 574	1344± 2073
hsa-miR-664a-5p[*]	385 ± 642	5546 ± 5985
hsa-miR-760[*]	221 ± 380	1646 ± 1422
hsa-miR-766-5p[*]	43 ± 75	1151 ± 1935
hsa-miR-92b-5p	459 ± 785	2829 ± 2512

[1]Values are means ± SD (n = 3 subjects).
[2]Human (*hsa*) and chicken (*gga*) sequences are identical, except for occasional extensions by one nucleotide.
*$0.0001 < p < 0.01$ by paired-sample t test with multiple test correction.

The following supplementary tables were uploaded as separate files in Excel format.

Supplementary Table 5. mRNA expression in PBMCs before (t = h) and after (t = 9 h) consumption of 4 hard-boiled eggs in healthy adults (threshold used: 1.5-fold change).

Supplementary Table 6. KEGG pathways and GO families in human PBMCs altered by the consumption of 4 hard-boiled eggs in healthy adults (9 h after compared to before egg consumption).
